# Construction and Evaluation of a Korean Native Microbial Consortium for the Bioremediation of Diesel Fuel-Contaminated Soil in Korea

**DOI:** 10.3389/fmicb.2018.02594

**Published:** 2018-10-30

**Authors:** Yunho Lee, Sang Eun Jeong, Moonsuk Hur, Sunghwan Ko, Che Ok Jeon

**Affiliations:** ^1^Department of Life Science, Chung-Ang University, Seoul, South Korea; ^2^Microorganism Resources Division, National Institute of Biological Resources, Incheon, South Korea; ^3^Ecophile Co., Ltd., Seoul, South Korea

**Keywords:** microbial consortium, bioremediation, diesel fuel, hydrocarbons, Korea

## Abstract

A native microbial consortium for the bioremediation of soil contaminated with diesel fuel in Korea was constructed and its biodegradation ability was assessed. Microbial strains isolated from Korean terrestrial environments, with the potential to biodegrade aliphatic hydrocarbons, PAHs, and resins, were investigated and among them, eventually seven microbial strains, *Acinetobacter oleivorans* DR1, *Corynebacterium* sp. KSS-2, *Pseudomonas* sp. AS1, *Pseudomonas* sp. Neph5, *Rhodococcus* sp. KOS-1, *Micrococcus* sp. KSS-8, and *Yarrowia* sp. KSS-1 were selected for the construction of a microbial consortium based on their biodegradation ability, hydrophobicity, and emulsifying activity. Laboratory- and bulk-scale biodegradation tests showed that in diesel fuel-contaminated soil supplemented with nutrients (nitrogen and phosphorus), the microbial consortium clearly improved the biodegradation of total petroleum hydrocarbons, and all microbial strains constituting the microbial consortium, except for *Yarrowia* survived and grew well, which suggests that the microbial consortium can be used for the bioremediation of diesel fuel-contaminated soil in Korea.

## Introduction

Soil contamination with toxic compounds has become a great environmental concern in recent years because toxic compounds in soil and groundwater are a threat to both human health and nature (Chen et al., 2015). In particular, an accidental oil spill leads to the release of large quantities of petroleum into the environment, and subsequently perturbs environmental ecosystems tremendously (Khamforoush et al., 2013; Macaulay and Rees, 2014). Bioremediation, which relies on microbiological processes, has proven to be a non-disruptive, cost-effective, and highly efficient approach to remove organic pollutants, particularly compared to other physico-chemical approaches (Tyagi et al., 2011; Fathepure, 2014; Zhao et al., 2017).

Many diverse microorganisms that can be used for the bioremediation of contaminated soil have been isolated from terrestrial habitats worldwide (Jeon et al., 2003; Das and Chandran, 2011; Fierer et al., 2012; Jin et al., 2012; Bardgett and van der Putten, 2014; Shekhar et al., 2015; Ismaeil et al., 2018; Roy et al., 2018). However, the accessibility and availability of biological resources obtained from abroad is currently limited due to stringent international law and national legislation, such asthe Convention on Biological Diversity (CBD^1^) and the Nagoya Protocol on Access and Benefit Sharing (ABS^2^). Therefore, many efforts have been made to isolate, preserve, and characterize biological resources, to overcome these legal issues in all countries (Overmann, 2015; Overmann and Scholz, 2017).

Most contaminated sites are generally contaminated with multiple pollutants rather than a single type, and harbor a variety of different environmental conditions for biological activity (Andreoni and Gianfreda, 2007). Therefore, bioremediation using a single type of microorganism often results in failure, due to low biodegradability, adaptability, and viability of the applied microorganisms in a contaminated site with diverse environmental conditions (Tyagi et al., 2011; Rayu et al., 2012; Herrero and Stuckey, 2015). To accomplish successful bioremediation, many issues, including the types of organic compound present, the use of appropriate biodegrading microorganisms and their biodegradation properties, and diverse environmental factors such as water content, temperature, pH, and heavy metal content should be addressed (Gandolfi et al., 2010; Jeon and Madsen, 2013), but are not easily resolved due to their complexity. However, these limitations may be easily overcome by the application of a microbial consortium consisting of multiple strains with diverse biodegradation abilities and physiological properties that ensure survival in a contaminated site with diverse environmental conditions (Rahman et al., 2002; Thompson et al., 2005; Wu et al., 2012; M′rassi et al., 2015; Ai-Kindi and Abed, 2016; Li et al., 2016; Gurav et al., 2017). In this study a native microbial consortium, complying with international law and national legislation for biological resources, was constructed using microbial strains that were isolated from Korea, for the bioremediation of diesel fuel-contaminated soil in Korea. The ability of the microbial consortium to biodegrade diesel fuel compounds was assessed in laboratory- and bulk-scale systems.

## Materials and Methods

### Microbial Strains for the Construction of a Native Microbial Consortium

To construct a native microbial consortium for the bioremediation of diesel fuel-contaminated soil in Korea, we obtained a list of candidate microorganisms potentially capable of degrading diesel fuel compounds, including aliphatic hydrocarbons, polycyclic aromatic hydrocarbons (PAH), or resins, that were isolated from terrestrial environments in Korea, through a literature search of research articles, patents, and other reference materials. As many candidate diesel fuel compound-degrading microbial strains were collected as possible from authors or culture collection centers.

The ability of the collected microbial strains to biodegrade diesel fuel compounds was assessed based on their growth when using *n*-hexadecane, naphthalene, or crude oil as a sole carbon source. Three cotton-plugged 250 mL Erlenmeyer flasks containing 50 mL of minimal salt basal (MSB; Stanier et al., 1966) broth were prepared and 0.25 g of naphthalene pellets (Sigma-Aldrich, United States), 0.5 mL of hexadecane (Sigma-Aldrich, United States), or 0.5 mL of crude oil (Sigma-Aldrich, United States) were directly added into each flask. Cells of microbial strains grown in R2A broth (BD, United States) at their optimum temperatures with shaking (200 rpm) were inoculated (1%, v/v) into each flask. The flasks were incubated with shaking (200 rpm) at their optimum temperatures. After 2 days of incubation, the growth of the microbial strains was assessed by measuring their optical densities at 600 nm (OD_600_). In addition, the biodegradation ability of the collected microbial strains was also assessed based on their growth on MSB agar supplied with *n*-hexadecane, naphthalene, or crude oil as a sole carbon and energy source. Collected microbial strains were cultured in MSB broth or on MSB agar without the supplementation of the carbon sources as a negative control.

### Hydrophobicity and Emulsifying Activity

Cell surface hydrophobicity of microbial strains with good biodegradation ability was evaluated through a bacterial adherence to hydrocarbons (BATH) assay with some modifications (Sorongon et al., 1991). In brief, cells of microbial strains grown in R2A broth were washed and resuspended in phosphate-buffered saline (PBS; 137 mM NaCl, 2.7 mM KCl, 10 mM Na_2_HPO_4_, 2 mM KH_2_PO_4_, pH 7.2). Three milliliters of the microbial suspension with approximately 2 × 10^8^ cells/mL in PBS was vigorously mixed with 1 mL of *n*-hexadecane. The mixtures were left to stand for 30 min at room temperature (RT) for separation into aqueous and organic phases, and the OD_600_ of the aqueous phase was measured. Hydrophobicity (BATH%) of microbial strains was calculated as (a - b)/a × 100, where *a* is the OD_600_ of the microbial suspension in PBS and *b* is the OD_600_ of the aqueous phase after partitioning (Sorongon et al., 1991; Zita and Hermansson, 1997).

The emulsifying activity of microbial strains was assessed according to a previously described method (Shahaliyan et al., 2015), with some modifications. In brief, cells of microbial strains grown in R2A broth were washed and resuspended in PBS. Three milliliters of the microbial suspension with approximately 2 × 10^8^ cells/mL in PBS were vigorously mixed with an equal volume of crude diesel and left for 24 h at RT. The emulsification index (*E*_24_) of microbial strains was calculated as the height of emulsified layer/height of total mixture × 100. The cell surface hydrophobicity and emulsifying activity of the microbial strains were expressed as the mean values of duplicated experiments.

### Biodegradation of Diesel Fuel Compounds With a Microbial Consortium in a Laboratory-Scale Soil System

Soil was collected from a diesel fuel-contaminated area near a gas station in Seoul, Korea and transported to the laboratory. The soil was analyzed for particle size and content of total organic carbon, total nitrogen, and phosphorus at the National Instrumentation Center for Environmental Management of Seoul National University (NICEM, Korea). The soil was sieved through a 2 mm mesh to remove large particles, and then diesel fuel was spiked into the soil at approximately 2,000 mg diesel/kg-soil and the diesel-spiked soil was well mixed.

To prepare a microbial consortium, microbial strains with good biodegradation ability were separately cultured in R2A broth at 25°C for 3 days. The microbial cells were harvested by centrifugation at 10,000 *g* for 30 min at 4°C and washed with 0.85% (w/v) saline buffer. Cells of each bacterial strain were mixed at an equal proportion and yeast cells (*Yarrowia* sp. KSS-1) corresponding to approximately 1% of each bacterial strain were added to the bacterial cell mixture to constitute a final microbial consortium to be approximately 10^10^ cells/mL in 0.85% saline buffer. To assess ability of the microbial consortium to degrade diesel fuel in soil, four laboratory test sets with 1 kg soil per sample were prepared in triplicate using the diesel-spiked soil: untreated; treated with 140 mg/kg-soil of (NH_4_)_2_SO_4_ and 14 mg/kg-soil of KH_2_PO_4_ as nutrients; treated with the microbial consortium; and treated with both nutrients and the microbial consortium. The microbial consortium was inoculated into the diesel-spiked soil to a final concentration of approximately 10^7^ cells/g-soil. The four test sets were incubated at RT in the dark and soil samples for the analysis of total petroleum hydrocarbons (TPHs) and microbial community composition and abundance were taken at 0 (prior to dispensing into four test bottles), 3, 5, 10, and 15 days of incubation.

### Analysis of the Bacterial Community Present in Lab-Scale Soil During the Test Period

Total genomic DNA from 0.5 g of soil sample was extracted using the FastDNA Spin kit for soil (MPbio, United States), according to the manufacturer’s instructions. The V3–V4 regions of bacterial 16S rRNA genes from the total genomic DNA were PCR-amplified and sequenced using a Illumina Miseq platform (Roche, Germany) at Macrogen (Korea) after pooling the PCR products, as described previously (Miran et al., 2017). The Illumina Miseq sequencing data of the pooled PCR products were sorted based on their unique barcode sequences and then the barcode and adapter sequences were trimmed using Scythe (Catchen et al., 2011; Buffalo, 2014) and Sickel software (Joshi and Fass, 2011). The sorted sequencing data were processed and classified using Mothur software v. 1.39.5 following MiSeq SOP^3^. In brief, low-quality sequencing reads with ambiguous base calls (“N”) and those shorter than 200-bp were removed and potential chimeric sequences were also excluded from the next analysis. Representative sequences of operational taxonomic units that were clustered at 3% divergence were classified at the genus level against the Silva v128 database (Quast et al., 2013).

### Quantitative Real-Time PCR (qPCR) to Estimate the Number of Total Bacteria and *Yarrowia* sp.

The number of total bacteria and *Yarrowia* sp. in the soil of the four test sets during incubation were estimated using qPCR, as described previously (Lee et al., 2014). In brief, 100 ng of salmon sperm DNA (Sigma, United States) was added to 0.5 g of the soil samples as an internal standard, and then the total genomic DNA was extracted using the FastDNA Spin kit for soil. The primer pairs 340F (5′-CCT ACG GGA GGC AGC AGT-3′)/758R (5′-CTA CCA GGG TAT CTA ATC C-3′) and Yarr-F (5′-TCA ACA ACG GAT CTC TTG GC-3′)/Yarr-rev (5′-ATA CCA TAC CGC GCA ATG TG-3′), targeting bacterial 16S rRNA genes and rRNA internal transcribed spacers (ITS) of *Yarrowia* sp., respectively, were used for the qPCR quantification of total bacteria and *Yarrowia* sp., respectively (Juck et al., 2000; Vogel et al., 2017) and the qPCR amplifications for bacteria and yeasts were conducted according to the PCR conditions described previously (Jung et al., 2014). Standard curves for the gene copy calculations of total bacteria and *Yarrowia* sp. were generated using the genomic DNAs extracted from a known number of *Pseudomonas* sp. AS1 and *Yarrowia* sp. KSS-1 cells. Sample-to-sample variation caused by differences in genomic DNA extractions and qPCR efficiencies were normalized based on qPCR results using primers Sketa2-F (5′-GGT TTC CGC AGC TGG G-3′)/Sketa2-R (5′-CCG AGC CGT CCT GGT CTA-3′), targeting the rRNA ITS region 2 of the salmon sperm DNA, as described previously (Haugland et al., 2005). The absolute abundances of bacterial strains composing the microbial consortium in the soil of the four test sets were estimated by multiplying the relative abundances of the genera that were classified at the genus level and the gene copy numbers of bacterial 16S rRNA genes that were obtained by the qPCR quantification, as described previously (Lee et al., 2015).

### Biodegradation of TPHs by the Microbial Consortium in a Bulk-Scale Soil System

A bulk-scale biodegradation test was conducted at Ecophile Co., Ltd. (Korea) to evaluate the field application of the constructed microbial consortium for the bioremediation of diesel fuel-contaminated soil. Two samples of 100 metric tons of diesel fuel-contaminated soil with approximately 2,300 mg TPHs/kg-soil were established: untreated and treated with nutrients (800 mg/kg-soil of (NH_4_)_2_SO_4_ and 80 mg/kg-soil of KH_2_PO_4_) and the microbial consortium, which was inoculated into the soil to a final concentration of approximately 1.1 × 10^6^ cells/g-soil. Tilling and watering were conducted every 3 days to ensure moisture content and aeration of the contaminated soil. Soil samples for the TPH analysis were taken from three different parts of diesel fuel-contaminated soil after 4, 7, 10, 14, and 17 days of incubation.

Three grams of each soil sample was collected in glass bottles and 10 mL of dichloromethane containing 1-chloronaphthalene (100 mg/L) was added into the soil samples as an internal standard. The glass bottles were vigorously vortexed for at least 5 min and then subjected to ultrasound for 30 min. The organic phase containing TPHs was obtained by centrifugation at 3,000 *g* for 5 min and TPHs were analyzed with gas chromatography (GC) using an HP 7890B gas chromatograph coupled to a flame ionization detector (Agilent, United States). One microliter was injected with an auto-sampler into an HP-5 column (30 m length, 0.32 μm inner diameter, 0.25 μm film thickness; J & W Scientific) in a split mode (5:1) and nitrogen was used as a carrier gas (3.5 mL/min). The injection temperature was 300°C and the oven temperature was held at 40°C for 0.5 min and increased by 10°C per min to 150°C and then at 15°C per min to a final temperature of 290°C, where it was held for 5 min. The amount of TPH in soil was calculated based on the sum of the peak areas in the GC chromatograms.

## Results and Discussion

### Selection of Korean Native Microbial Strains With the Ability to Biodegrade Diesel Fuel Compounds

To construct a native microbial consortium for the bioremediation of diesel fuel-contaminated soil in Korea, all microbial strains isolated from terrestrial environments in Korea with the potential to biodegrade aliphatic hydrocarbons, PAH, or resins were found using an wide-ranging literature search. A total of 116 microbial strains were identified as initial candidates capable of biodegrading diesel fuel compounds, and among them, only 18 microbial strains were available for use in the next biodegradation test (Table 1): most of the candidate microbial strains had either been lost or not preserved following their studies.

**Table 1:** List of microbial strains used in this study and their growth in minimal salt basal (MSB) broth or on MSB agar using hexadecane (for aliphatic hydrocarbon degraders), naphthalene (for PAH degraders), and crude oils (for resin degraders) as a sole carbon and energy source.

Test compound	Strain^$^	Growth		Reference/source
		MSB broth^∗^	MSB agar^†^	
Hexadecane	***Acinetobacter* sp. DR1**	++	+	Kang et al., 2011
	*Pseudomonas* sp. DR2	+	±	Song et al., 2008
	***Yarrowia* sp. KSS-1**	++	+	Ecophile Co., Ltd.
	***Corynebacterium* sp. KSS-2**	+ + +	+	Ecophile Co., Ltd.
	*Microbacterium* sp. KSS-7	+	±	Ecophile Co., Ltd.
	*Nocardia* sp. H17-1	±	±	Baek et al., 2004
Naphthalene	*Rhodococcus* sp. KOL-1	-	+	Ecophile Co., Ltd.
	***Pseudomonas* sp. AS1**	+ + +	+	Kang et al., 2006
	*Gordonia* sp. H37	±	±	Kwon et al., 2015
	*Arthrobacter* sp. S49	±	±	Kwon et al., 2015
	*Brevibacterium* sp. S47	±	±	Kwon et al., 2015
	*Burkholderia cepacia* 2A-12	±	±	Kim et al., 2003
	***Pseudomonas* sp. Neph5**	+	+	KNIBR^‡^
	*Sphingobium* sp. Phe1	±	±	KNIBR^‡^
	*Novosphingobium* sp. Phe9	±	±	KNIBR^‡^
Crude oil	***Micrococcus* sp. KSS-8**	+ + +	+	Ecophile Co., Ltd.
	*Bacillus* sp. KOL-4	++	±	Ecophile Co., Ltd.
	***Rhodococcus* sp. KOS-1**	++	+	Ecophile Co., Ltd.

*^∗^Microbial cells were inoculated into MSB broth supplied with hexadecane, naphthalene, or crude oil and their growth was evaluated at OD_*600*_ after 2 days of incubation. Growth (OD_*600*_): >1.0, + + +; 0.5∼1.0, ++; 0.1–0.5, +; <0.1, ±. ^†^Hexadecane, naphthalene, or crude oil was supplied to microbial cells on MSB agar and their growth was evaluated by colony formation after 2 days of incubation. Growth: +, clear colony formation; ±, no or weak colony formation. ^$^Microbial strains highlighted in bold were selected for the construction of a microbial consortium.*

The biodegradation ability of the 18 microbial strains obtained through their authors or culture collection centers was assessed based on their growth in MSB broth and on MSB agar supplemented with *n*-hexadecane, naphthalene, or crude oil as a sole carbon and energy source (Table 1). Based on growth ability, three aliphatic hydrocarbon-degrading microbial strains, *Yarrowia* sp. KSS-1, *Corynebacterium* sp. KSS-2, and *Acinetobacter* sp. DR1 (Kang et al., 2011); two PAH-degrading microbial strains, *Pseudomonas* sp. AS1 and *Pseudomonas* sp. Neph5, and two resin-degrading microbial strains, *Rhodococcus* sp. KOS-1 and *Micrococcus* sp. KSS-8, were finally selected to construct a microbial consortium for the bioremediation of diesel fuel-contaminated soil in Korea (Table 1).

### Hydrophobicity and Emulsifying Activity of Selected Microbial Strains

**FIGURE 1:**
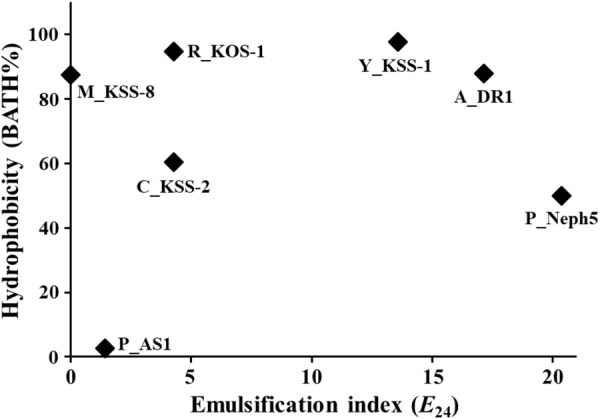
Cell surface hydrophobicity (BATH%) and emulsification index (*E*_24_) of seven microbial strains with a good ability to biodegrade diesel compounds. A_DR1, *Acinetobacter* sp. DR1; Y_KSS-1, *Yarrowia* sp. KSS-1; C_KSS-2, *Corynebacterium* sp. KSS-2; P_AS1, *Pseudomonas* sp. AS1; P_Neph5, *Pseudomonas* sp. Neph5; M_KSS-8, *Micrococcus* sp. KSS-8; and P_KOS-1, *Rhodococcus* sp. KOS-1.

Organic pollutants, including diesel fuel compounds, are generally highly hydrophobic and not very water-soluble, which increases their sorption to soil particles and decreases their bioavailability to microorganisms (Cerniglia, 1993). One approach to enhance the bioavailability of hydrophobic hydrocarbon compounds by microorganisms is to apply synthetic surfactants or biosurfactants to the contaminated soil (Sheng et al., 2010; Elliot et al., 2011; Shekhar et al., 2015). However, the use of synthetic surfactants can be sometimes unsuitable in bioremediation applications because they may have toxic effects on the environment or result in secondary pollution. This limitation can be solved by the use of microorganisms with high cell surface hydrophobicity or emulsifying activity from the production of surfactants (Gutierrez et al., 2013). Therefore, the cell surface hydrophobicity and emulsifying activity of seven microbial strains were assessed (Figure 1).

The BATH assay showed that all test strains except for *Pseudomonas* sp. AS1 displayed relatively high cell surface hydrophobicity of more than 50 BATH%. The emulsification activity test showed that *Pseudomonas* sp. Neph5, *Acinetobacter* sp. DR1, and *Yarrowia* sp. KSS-1 displayed relatively high emulsifying activity, with an *E*_24_ of more than 10, while *Rhodococcus* sp. KOS-1, *Corynebacterium* sp. KSS-2, *Pseudomonas* sp. AS1, and *Micrococcus* sp. KSS-8 displayed relatively low emulsifying activity, with an of *E*_24_ of less than 5. Strains Neph5 and AS1 had quite different cell surface hydrophobicity and emulsification index, although they both belong to the same genus of *Pseudomonas*. The hydrophobicity and emulsifying activity of microbial strains are known to improve biodegradation efficiency and rate by increasing microbial accessibility to hydrophobic organic pollutants (Sheng et al., 2010; Elliot et al., 2011). Strains KSS-1, DR1, and Neph5 had high hydrophobicity and emulsifying activity, suggesting that they may easily access hydrophobic diesel fuel compounds and effectively biodegrade them in contaminated soil.

Although strain AS1 exhibited low cell surface hydrophobicity and emulsifying activity, it was reported that it might be responsible for the degradation of PAH in contaminated soil (Kang et al., 2006, 2007). Emulsification indices of strains KSS-2, KSS-8, and KOS-1 were low, but their cell surface hydrophobicity was relatively high, which suggests that the cell surface hydrophobicity and emulsifying activity of microorganisms may be attributed to different mechanisms. The key microorganisms mainly responsible for bioremediation in contaminated sites are different depending on the specific contaminated site because adaptability and viability of the microorganisms applied are different depending on the contaminated site (Andreoni and Gianfreda, 2007; Das and Chandran, 2011; Shekhar et al., 2015). Therefore, the employment of multiple microorganisms capable of biodegrading the same organic pollutants increases the probability of successful bioremediation because it is more likely that at least one microorganism will show good adaptability and viability in a specific contaminated site. Although *Pseudomonas* sp. AS1 has low cell surface hydrophobicity and emulsifying activity, we used all seven microbial strains to construct a microbial consortium for bioremediation of diesel fuel-contaminated sites because the strain can effectively degrade hydrophobic diesel fuel compounds with the help of other biosurfactant-producing microorganisms.

### Effects of a Microbial Consortium and Nutrients on the Biodegradation of Diesel Fuel Compounds in a Lab-Scale Soil System

**FIGURE 2:**
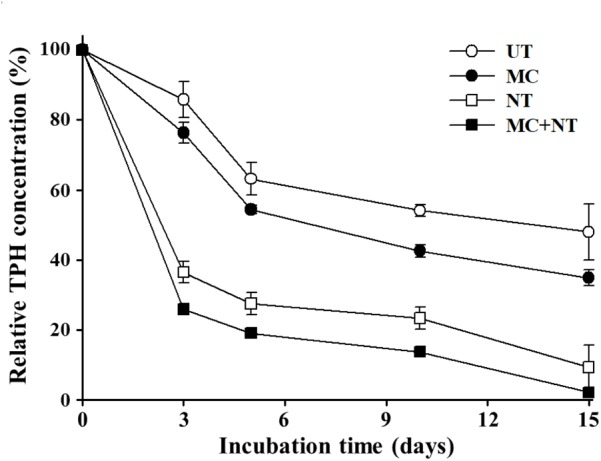
Effects of the microbial consortium and nutrients on the biodegradation of diesel fuel compounds in a laboratory-scale soil system. The initial concentration of total petroleum hydrocarbons (TPHs) was approximately 2,000 mg/kg soil and TPH concentrations in the soil throughout the incubation time were relatively expressed based on the TPH concentration in soil before treatment (day 0). The experiments were performed in triplicate and standard errors are indicated. UT, untreated; MC, treated with the microbial consortium; NT, soil treated with nutrients; MC + NT, treated with the microbial consortium and nutrients.

In order to investigate the effects of a microbial consortium on the biodegradation of diesel fuel compounds in soil, diesel fuel-contaminated soil was collected from a gas station and its soil properties were analyzed. The soil had a sandy loam type texture (12.3% clay, 12.1% silt, and 75.6% sand) with a neutral pH and contained ∼300 mg TPHs/kg soil, ∼340 mg total nitrogen/kg soil, and ∼250 mg phosphate (P_2_O_5_)/kg soil. The soil was additionally spiked with diesel fuel to a final concentration of ∼2,000 mg TPHs/kg soil and the effects of the microbial consortium and nutrients on the biodegradation of diesel fuel compounds in soil were assessed (Figure 2). The biodegradation test showed that TPH decrease occurred rapidly even in soil without treatment, which might be caused by TPH biodegradation by indigenous bacteria or TPH volatilization. Treatment with only nutrients (nitrogen and phosphorus) also improved the decrease of TPH and its effect was greater than the inoculation of the microbial consortium, which supports the notion that nutrient availability is a very important limiting factor with significant effects on the biodegradation of organic pollutants in environments (Jin et al., 2013) although it is known that many other factors such as biosurfactants, pH, moisture, and predators also largely influence pollutant bioremediation (Das and Chandran, 2011; Tyagi et al., 2011).

There have been many reports that the biodegradation of organic pollutants by indigenous microorganisms is promoted only by the supplementation of nutrients (Chang et al., 2010; Karamalidis et al., 2010; Ai-Kindi and Abed, 2016) and that treatment with nutrients can be more efficient than the supplement of exogeneous microorganisms because exogeneous microorganisms will sometimes fail to survive and grow in contaminated sites (Atlas, 1995; Margesin and Schinner, 2001; Bento et al., 2005; Thompson et al., 2005). However, the degradation test showed that the inoculation of a microbial consortium clearly improved the TPH decrease in the contaminated soil. In particular, the treatment of both the microbial consortium and nutrients together was shown to improve TPH biodegradation more than their single treatment. At 15 days of incubation, the TPH concentration decreased to a very low concentration (∼2.2%) with the treatment of microbial consortium and nutrients. These results suggest that the microbial consortium can efficiently bioremediate soil contaminated with diesel fuel.

Rahman et al. (2002) reported that a bacterial consortium consisting of five oil-degrading bacterial strains that were isolated from oil-contaminated soil samples degraded a maximum of 78% of Bombay High crude oil. Alisi et al. (2009) also reported that a tailored microbial consortium using ten bacterial strains from an indigenous microbial community showed about 75% reduction of TPHs in 42 days. Li et al. (2016) reported that a microbial consortium comprising five fungi and three bacteria biodegraded crude oil spilled in China’s Bohai Sea more efficiently than a single strain. These previous results also support the idea that reliable and efficient bioremediation of various environments contaminated with mixed organic compounds can be accomplished by the use of microbial consortia consisting of multiple microorganisms. In addition, these results suggest that both the application of a microbial consortium and of nutrients are necessary for more reliable and successful bioremediation of soil contaminated with organic pollutants.

### Abundance of Microbial Strains in Soil During the Laboratory-Scale Test Period

**FIGURE 3:**
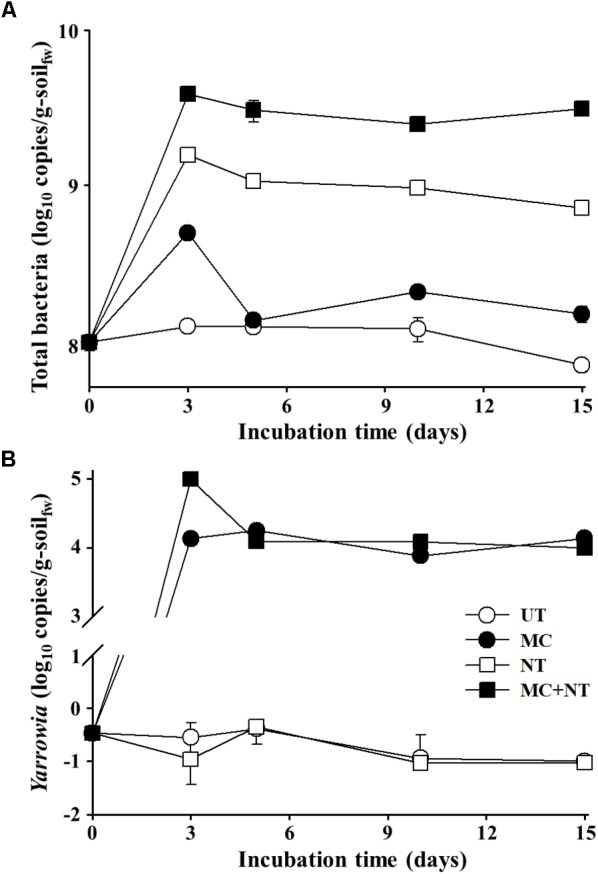
Estimated abundance of 16S rRNA gene copies of total bacteria **(A)** and rRNA internal transcribed spacer gene copies of *Yarrowia* **(B)** in the soil of four different test sets during incubation. The measurements were performed in triplicate using qPCR and standard errors are indicated. g-soil_fw_, gram-soil fresh weight; UT, untreated; MC, treated with the microbial consortium; NT, treated with nutrients; MC + NT, treated with the microbial consortium and nutrients.

The abundance of total bacteria and *Yarrowia* sp. KSS-1 constituting the constructed microbial consortium in the diesel fuel-spiked soil during the degradation test period was assessed using qPCR. The qPCR analysis targeting total bacteria showed that bacterial abundances depending on the treatments were relatively well-associated with the decrease of THP, depending on the treatments shown in Figure 3A. The bacterial abundance significantly increased even with the application of nureients only and this increase was greater than the increase observed by treatment with the microbial consortium alone. In particular, the treatment of both the microbial consortium and nutrients together clearly improved bacterial growth more than their single treatments during the degradation test period. These results suggest that nutrients might be an important limiting factor for bacterial growth in the contaminated soil. In addition, the qPCR analysis suggested that indigenous bacteria, as well as exogeneous bacteria, contributed to the biodegradation of diesel fuel compounds in the contaminated soil. The qPCR analysis targeting *Yarrowia* showed that *Yarrowia* cells were detected only from soil treated with the microbial consortium and the nutrient treatment did not improve the growth of *Yarrowia* unlike bacteria (Figure 3B), which suggests that *Yarrowia* cells might not grow in the contaminated soil.

**FIGURE 4:**
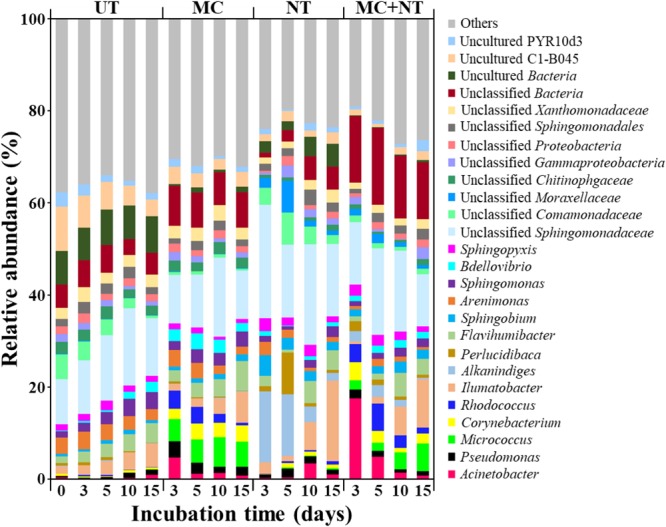
Bacterial taxonomic compositions at the genus level in the soil of four different test sets during incubation. The genera with less than 3% of relative abundances in all samples were categorized into “Others.” UT, untreated; MC, treated with microbial consortium; NT, treated with nutrients; MC + NT, treated with microbial consortium and nutrients.

**FIGURE 5:**
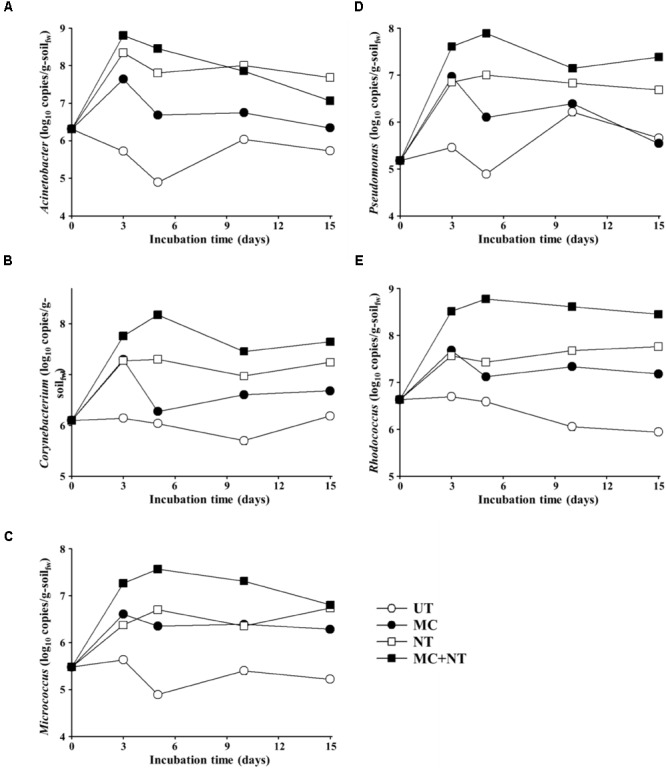
Estimated absolute abundances of *Acinetobacter* **(A)**, *Corynebacterium* **(B)**, *Micrococcus* **(C)**, *Pseudomonas* **(D)**, and *Rhodococcus* **(E)** during incubation of the soil of four different test sets. The absolute abundances (16S rRNA gene copy numbers) of bacterial genus groups were estimated by multiplication of the relative abundances shown in Figure 4 and the corresponding 16S rRNA gene copy numbers of total bacteria in Figure 3A. g-soil_fw_, gram-soil fresh weight; UT, untreated; MC, treated with the microbial consortium; NT, treated with nutrients; MC + NT, treated with the microbial consortium and nutrients.

To estimate the absolute abundances of bacterial strains constituting the microbial consortium in the contaminated soil samples, bacterial communities were investigated through Illumina Miseq sequencing analysis of 16S rRNA gene amplicons. The relative abundances of each bacterial genus group (Figure 4) were multiplied by the total copy numbers of bacterial 16S rRNA genes (Figure 3A). The absolute abundance of all bacterial genus groups constituting the microbial consortium was increased by all three treatments (Figure 5), which suggests that all bacterial strains can survive and grow well in the contaminated soil. In particular, only nutrient treatment increased the absolute abundance of all bacterial genus groups, suggests that the same bacterial genus groups corresponding to the bacterial strains constituting the microbial consortium were also present indigenously in the contaminated soil.

The abundance of *Acinetobacter* in soil treated only with nutrients was similar to the soil treated with nutrients and the microbial consortium together, which suggests that nutrient treatment promoted the growth of indigenous *Acinetobacter* members present in the contaminated soil, not the exogenous *Acinetobacter* strain. On the other hand, the abundance of *Rhodococcus, Pseudomonas, Corynebacterium*, and *Micrococcus* in soil treated with nutrients and microbial consortium together was clearly greater than those in soil treated only with nutrients, which suggests that nutrient treatment promoted the growth of exogenous *Rhodococcus, Pseudomonas, Corynebacterium*, and *Micrococcus* strains constituting the microbial consortium, and that they survived and grew well in the contaminated soil. However, the analysis showed that bacterial strains constituting the microbial consortium did not grow well in the contaminated soil without the supplementation of nutrients. Although a nutrient supply is very effective in promoting microbial activity in nutrient-depleted soil, it is not easy to supply nutrients with bioavailability efficiently to a contaminated soil, especially in *in situ* soil bioremediation. Thus, combined bioaugmentation processes with other approaches to stimulate microbial activity in soil such as plant exudates have been suggested as efficient bioaugmentation processes (Zhao et al., 2017).

### Bioremediation of TPHs by the Microbial Consortium in a Bulk-Scale Soil System

**FIGURE 6:**
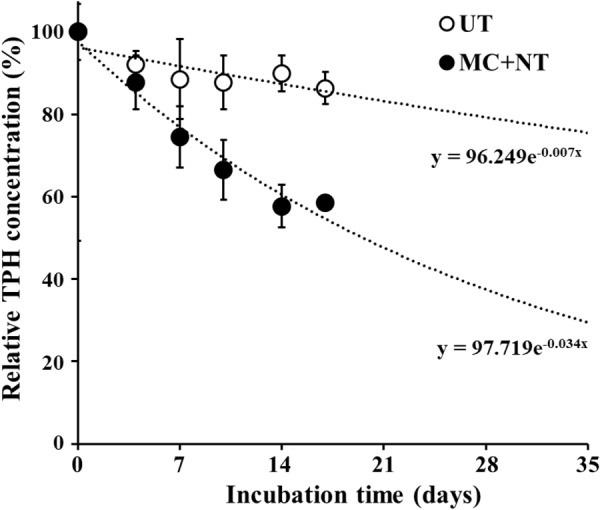
Effects of the microbial consortium and nutrients on the bioremediation of diesel fuel-contaminated soil in a bulk-scale system (100 metric tons each). The initial concentration of total petroleum hydrocarbons (TPHs) was approximately 2,300 mg/kg-soil and TPH concentrations in soil over incubation time were relatively expressed based on the TPH concentration at 0 day. TPH analysis was performed using soil samples taken from three different parts of bulk-scale soil. UT, untreated; MC + NT, treated with the microbial consortium and nutrients. TPH concentrations after 17 days in UT and MC + NT soil were extrapolated using the equations, marked below the lines.

TPH biodegradation by the constructed microbial consortium was assessed in a bulk-scale diesel fuel-contaminated soil system. Based on the results of biodegradation tests at laboratory-scale, microbial strains constituting the microbial consortium together with nutrients were applied to diesel fuel-contaminated soil in bulk-scale. The application of the microbial consortium and nutrients to the contaminated soil clearly improved TPH biodegradation compared to untreated soil (Figure 6). After14 days of incubation, TPHs decreased to 42.3% of their initial concentration in soil treated with the microbial consortium and nutrients and the decrease was clearly greater than the 10.1% decrease observed in the bulk scale soil without treatment. These results suggest that a combination of bioaugmentation using exogeneous microbial consortium and biostimulation using nutrients (nitrogen and phosphorus) can effectively clean up polluted environments contaminated with organic compounds, as shown in previous reports (Kim et al., 2005; Jimenez et al., 2006; Garcia-Blanco et al., 2007; Karamalidis et al., 2010; Zhao et al., 2017).

## Conclusion

A native microbial consortium using seven microbial strains with a good ability to biodegrade aliphatic hydrocarbons, PAH, or resins, which were isolated from terrestrial environments in Korea, was constructed for the bioremediation of diesel fuel-contaminated soil in Korea. The application of the constructed microbial consortium with nutrients (nitrogen and phosphorus) clearly improved bioremediation of diesel fuel-contaminated soil in laboratory- and bulk-scale experiments. The microbial consortium of this study, which complies with international law and national legislation for biological resources, will be useful to effectively clean up polluted environments contaminated with organic compounds in Korea.

## Author Contributions

YL initiated the project, performed the experiments, and wrote the manuscript. SJ and MH analyzed the metagenomic data. SK designed the bulk-scale soil experiments and interpreted the results. CJ designed the experiments and revised the manuscript.

## Conflict of Interest Statement

The authors declare that the research was conducted in the absence of any commercial or financial relationships that could be construed as a potential conflict of interest.

## Footnotes

1 https://www.cbd.int/

2 http://www.cbd.int/abs/

3 https://www.mothur.org/wiki/MiSeq_SOP
